# Investigation of the TeO_2_/GeO_2_ Ratio on the Spectroscopic Properties of Eu^3+^-Doped Oxide Glasses for Optical Fiber Application

**DOI:** 10.3390/ma15010117

**Published:** 2021-12-24

**Authors:** Magdalena Lesniak, Jakub Zeid, Bartłomiej Starzyk, Marcin Kochanowicz, Marta Kuwik, Jacek Zmojda, Piotr Miluski, Agata Baranowska, Jan Dorosz, Wojciech Pisarski, Joanna Pisarska, Dominik Dorosz

**Affiliations:** 1 Faculty of Materials Science and Ceramics, AGH University of Science and Technology, Av. A. Mickiewicza 30, 30-059 Krakow, Poland; jakub.zeid@o2.pl (J.Z.); starzyk@agh.edu.pl (B.S.); ddorosz@agh.edu.pl (D.D.); 2 Faculty of Electrical Engineering, Bialystok University of Technology, 45D Wiejska Street, 15-351 Bialystok, Poland; m.kochanowicz@pb.edu.pl (M.K.); j.zmojda@pb.edu.pl (J.Z.); p.miluski@pb.edu.pl (P.M.); doroszjan@pb.edu.pl (J.D.); 3 Institute of Chemistry, University of Silesia, 9 Szkolna Street, 40-007 Katowice, Poland; marta.kuwik@ud.edu.pl (M.K.); wojciech.pisarski@us.edu.pl (W.P.); joanna.pisarska@us.edu.pl (J.P.); 4 Faculty of Mechanical Engineering, Bialystok University of Technology, 45C Wiejska Street, 15-351 Bialystok, Poland; a.baranowska@pb.edu.pl

**Keywords:** oxide glasses, TeO_2_/GeO_2_ molar ratios, DSC, Eu^3+^ structural and spectroscopic probe, structure, PLE, PL, PSB, R ratio, decay time

## Abstract

This study presented an analysis of the TeO_2_/GeO_2_ molar ratio in an oxide glass system. A family of melt-quenched glasses with the range of 0–35 mol% of GeO_2_ has been characterized by using DSC, Raman, MIR, refractive index, PLE, PL spectra, and time-resolved spectral measurements. The increase in the content of germanium oxide caused an increase in the transition temperature but a decrease in the refractive index. The photoluminescence spectra of europium ions were examined under the excitation of 465 nm, corresponding to ^7^F_0_ → ^5^D_2_ transition. The PSB (phonon sidebands) analysis was carried out to determine the phonon energy of the glass hosts. It was reported that the red (^5^D_0_ → ^7^F_2_) to orange (^5^D_0_ → ^7^F_1_) fluorescence intensity ratio for Eu^3+^ ions decreased from 4.49 (Te0Ge) to 3.33 (Te15Ge) and showed a constant increase from 4.58 (Te20Ge) to 4.88 (Te35Ge). These optical features were explained in structural studies, especially changes in the coordination of ^[4]^Ge to ^[6]^Ge. The most extended lifetime was reported for the Eu^3+^ doped glass with the highest content of GeO_2_. This glass was successfully used for the drawing of optical fiber.

## 1. Introduction

Mid-infrared (MIR) lasers have received significant attention in the past because of their applications, e.g., eye-safe laser radar, monitoring air pollution, military, remote sensing, and surgery [1]. Up to now, the mid-infrared emission of active ions has been mainly focused on germanate [2], tellurite [3], fluoride [4], and chalcogenide [5] glasses. The optical properties of rare-earth (RE) ions have an important influence on mid-infrared applications. They are related to the host matrix’s phonon energy, the solubility of lanthanides ions, and the ligand field around rare-earth ions. Reducing the host glass phonon is a condition to achieve high-efficiency mid-infrared luminescence [6]. Among glass hosts, heavy metal oxides (HMO) glasses and especially the tellurite and germanate glasses have drawn growing interest as alternative hosts for mid-infrared emitting optical centers [7]. Tellurite glasses offer a wide transmission window from 0.4 to 6.5 µm, low maximum phonon energy (~750 cm^−1^), high rare-earth solubility, T_g_ (~350 °C), and high refractive indices (~2) and nonlinear coefficients 20–50 × 10^−20^ m^2^/W [8,9]. Due to these features, tellurite glasses might be used as waveguides or gain media for fiber lasers, Raman gain, fiber optical amplifiers, and broadband laser sources through supercontinuum (SC) generation [9]. Despite numerous advantages of tellurite glasses, their optical properties and thermal stability need to be optimized [10,11].

Compared with tellurite glasses, germanate glasses (~850 cm^−1^) have better thermal stability (due to the strong interionic forces between Ge^4+^ and O^−2^ ions), chemical durability, and higher resistance to laser damage threshold [12]. Barium gallo-germanate (BGG) glasses offer exceptionally high rare-earth solubility, strong mechanical strength, and NIR transparency [13]. Moreover, BGG glass presents the possibility to draw optical fibers. Wen et al. [14] reported the fabrication of BGG glass single-mode fibers doped with 1.8 mol% Tm^3+^ and a multi longitudinal-mode fiber laser at 1.95 μm. Tm^3+^/Ho^3+^ co-doped BGG glass and double-clad optical fiber for emission above 2 µm were presented by Kochanowicz et al. [15].

Moreover, tellurite and germanate glasses have also been investigated as suitable hosts for metal or semiconductor nanoparticles (NPs) [16,17,18,19]. For example, the 3.9 μm and 4.1 μm mid-infrared fluorescence emissions were analyzed in Ho^3+^/Yb^3+^/AgNPs doped TeO_2_–ZnO–WO_3_–La_2_O_3_–BaF_2_ glass system [16]. The infrared and up-conversion emissions of Er^3+^/Yb^3+^/Ce^3+^ co-doped tellurite glasses with copper nanoparticles (NPs) were also studied [17]. The obtained results have shown that the presence of the CuNPs enhanced Er^3+^ emission at 1.55 μm and increased the lifetime of the Er^3+^:^4^I_13/2_→^4^I_15/2_ transition by 50% [17]. The gains enhancement and an increase in the emitted intensity of RE ions because of the presence of the nanoparticles were observed in the germaniate systems [18,19]. The presence of the Ag-NPs led to 500% gain enhancement in the Bi_2_O_3_-GeO_2_ system doped with Tm^3+^ [18]. The enhanced infrared-to-visible frequency upconversion up to 100% in Yb^3+^/Er^3+^ co-doped Bi_2_O_3_-GeO_2_ glasses with 0.05 wt% concentration of AgNO_3_ was reported by Kassab et al. [19].

The tellurite-germanate glasses combine the advantages of both tellurite and germanate glasses. The influence of GeO_2_ content on thermal stability, structure, and spectroscopic feature of various tellurite glass systems was analyzed [20,21,22]. The authors presented the development of the tellurite-germanate optical fiber amplifiers [23,24,25], upconversion glass-fiber lasers [26], and phosphors [27]. GeO_2_ (0, 5, 15, 30, and 60 in mol%) was introduced in the Yb^3+^ -doped tellurite glass in the TeO_2_–ZnO–Na_2_O–GeO_2_-Yb_2_O_3_ system by Zhang et al. [28]. In this study, the effect of germanium dioxide content on the emission of Yb^3+^ ions was investigated. When the germanium dioxide concentration increased to 30 mol%, the absorption and emission cross-sections increased to 1.62 pm^2^ and 0.88 pm^2^, and then decreased with increasing GeO_2_ concentration to 60 mol%

Based on analysis of the Raman spectra, a detailed structural study was presented in a broad composition range of GeO_2_ (0, 20, 40, 60, and 100 mol%) in a TeO_2_–GeO_2_ system [29]. The various molar ratios of TeO_2_/GeO_2_ from 0.06 to 17 were reported in the ternary TeO_2_–GeO_2_–Ga_2_O_3_ system [30]. The tellurite glasses in xGeO_2_–TeO_2_–K_2_O–Na_2_O–Nb_2_O_5_–ZnO–Er_2_O_3_ (x = 0, 10, 20, 50, and 70 mol%) for 1.5 µm fiber and planar amplifiers were investigated by Zhao et al. [31].

It is seen that tellurite-germanate glasses can be considered as prospective hosts for RE doping and optical fiber drawing. However, a detailed analysis of that system has not already been published. This paper proposed thermal, structural, and spectroscopic characterization of the multicomponent TeO_2_-based system with various TeO_2_/GeO_2_ molar ratios. To perform luminescent studies, glasses have been doped with europium ions, including PLE, PL, and decay curves analysis. Additionally, based on structure investigations, the changes in the local environment of Eu^3+^ ions were explained. According to the decay time results of Eu^3+^ ions, one glass composition was selected, which was drawn into fiber. This proved that glass with the highest content of germanium dioxide has excellent thermal and fiberisation properties, i.e., optical (luminescent), and can be used as an active core in optical fibers.

## 2. Materials and Methods

Tellurite and tellurite-germanate glasses with general formula (64.60-x)TeO_2_-xGeO_2_-35(Ga_2_O_3_-ZnO-BaO-Na_2_O)-0.4Eu_2_O_3_, in mol% (where x = 0, 5, 10, 15, 20, 25, 30, and 35) were prepared by the melt-quenching technique using metal oxides of high purity 99.99%, Sigma-Aldrich, St. Louis, MI, USA. The glasses samples were denoted according to the GeO_2_ amount (Te0Ge, Te5Ge, Te10Ge, Te15Ge, Te20Ge, Te25Ge, Te30Ge, and Te35Ge). Each batch of 10 g was melted in a non-covered Pt/Ir crucible to 1100 °C with a 10 °C/min in ambient air. After reaching the maximum temperature, the melt was cast onto a preheated stainless steel and next annealed at 10 °C below the transformation temperature of 24 h. The annealed samples were divided into two parts. The first one was cut and polished (15 mm × 10 mm × 2 mm) for refractive index, Raman, PLE (photoluminescence excitation), PL (photoluminescence) spectra, and time-resolved spectral measurements. The second part was ground to the powder form for DSC (differential scanning calorimetry), XRD (X-ray diffraction), and MIR (mid-infrared) spectra investigations.

X-ray diffraction studies were conducted using the X’Pert Pro X-ray diffractometer (PANalytical, Almelo, The Netherlands) with Cu K_α1_ radiation (λ = 1.54056 Å) in the 2θ range of 10–90°. The thermal investigation, i.e., glass transition (T_g_) and crystallization (T_c_) temperature measurements, was carried out on Jupiter DTA STA 449 F3 thermal analyzer (NETZSCH Thermal Analysis, Selb, Germany). The glasses’ mid-infrared (MIR) absorption spectra were measured using the Fourier spectrometer (Bruker Optics-Vertex70V, Rheinstetten, Germany) at 128 scans, and the resolution of 4 cm^−1^. Raman spectra of glasses were recorded using a LabRAM HR spectrometer (HORIBA Jobin Yvon, Palaiseau, France). The excitation wavelength of 532 nm was used, and the diffraction grating was 1800 lines/mm. The MIR and Raman spectra were decomposed using Fityk software (0.9.8 software, open-source (GPL2+)) into Gauss maxima. The refractive index of all samples was measured at 632.8 nm by using a Metricon Model 2010/M prism coupler. The photoluminescence excitation (PLE), photoluminescence (PL) spectra, and luminescence decays were recorded at room temperature on a Horiba Jobin Yvon FluoroMax-4 spectrofluorimeter (Horiba Jobin Yvon, Longjumeau, France) supplied with a 150 W Xe lamp. The PLE and PL spectra were recorded with ±0.1 nm resolution and decay curves with ± 0.2 µs accuracies. The Eu^3+^ doping the glasses allowed analyzing the R (fluorescence intensity ratio), defined as the quotient of the ^5^D_0_ → ^7^F_2_ (red) to ^5^D_0_ → ^7^F_1_ orange intensity bands. The ^5^D_0_ → ^7^F_2_ emission is derived from the magnetic dipole transition, called a hypersensitive one. In the last step, the glass fiber was manufactured using SG controls drawing tower. Glass preform was fed into the tube furnace with 2–3 mm/min speed. The required diameter was regulated by the drum and the feed speed of the fiber preform. The drawing temperature was in the range of 700–800 °C.

## 3. Results

### 3.1. Glass Forming Ability (GFA)

The formation of the TeO_2_-based and tellurite-germanate glass systems has been intensively studied [32,33,34,35,36,37,38]. It might be found in the literature that the GFA of the ternary TeO_2_–GeO_2_–ZnO system is up to 80 mol% of GeO_2_ [32].

Other studies reporting the tellurite-germanate systems included: TeO_2_–GeO_2_–Ga_2_O_3_–Pr_6_O_11_ [33], GeO_2_−TeO_2_−K_2_O−Nb_2_O_5_−La_2_O_3_−Tm_2_O_3_ [34], GeO_2_−TeO_2_−Nb_2_O_5_−YF_3_−HoF_3_−ErF_3_−YbF_3_ [35], TeO_2_–ZnO–Na_2_CO_3_–GeO_2_–Er_2_O_3–_Ho_2_O_3_ [36], TeO_2_–GeO_2_–ZnO–BaO [37], and Bi_2_O_3_-80-TeO_2_–B_2_O_3_–GeO_2_ [38]. Its glass-forming ability enhanced considerably upon the addition of modifier oxides and heavy metal oxides [27,33,34,35,36,37,38].

**Figure 1:**
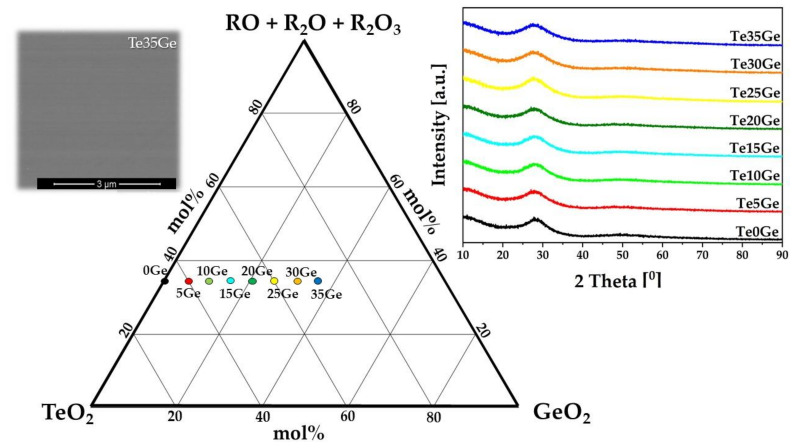
Glass-formation region of TeO_2_/GeO_2_–Ga_2_O_3_-BaO-ZnO-Na_2_O-Eu_2_O_3_ system at 1100 °C (in mol%). Diffraction patterns of glasses and SEM photo for Te35Ge glass (inset).

The glass-forming region of the TeO_2_–GeO_2_–Ga_2_O_3_–BaO–ZnO–Na_2_O–Eu_2_O_3_ system was obtained to our knowledge for the first time. The glass-forming domain of the tellurite and tellurite-germanate glasses was plotted in the TeO_2_–GeO_2_–RO + R_2_O + R_2_O_3_ diagram as shown in Figure 1 (where RO = BaO + ZnO; R_2_O = Na_2_O, and R_2_O_3_ = Ga_2_O_3_, Eu_2_O_3_). The thermal stable bulk glass samples were observed for the series glasses in the (64.60 − x)TeO_2_–xGeO_2_–35(Ga_2_O_3_–ZnO–BaO–Na_2_O)-0.4Eu_2_O_3_ system (in mol%) at 1100 °C, where x = 0, 5, 10, 15, 20, 25, 30, 35. All glassy samples were transparent and homogeneous. In the range of 2θ from 20° to 40°, a broad diffraction hump (inset in Figure 1—left side—diffraction patterns) might be observed. This is characteristic of amorphous materials [39]. The phase separation was not observed in all glasses (inset in Figure 1—right side—SEM photo for Te35Ge glass).

### 3.2. Thermal Properties and Refractive Indexes

#### DSC Curves of Glasses

**Figure 2:**
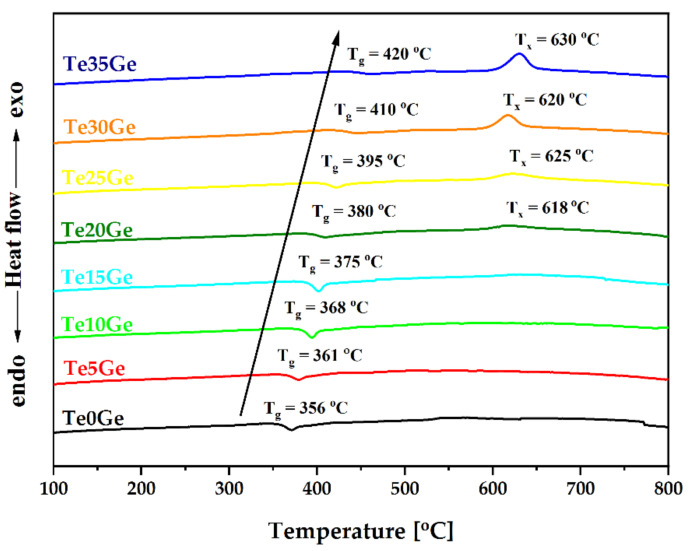
DSC curves of Eu^3+^-doped glasses with various TeO_2_/GeO_2_ molar ratios.

The thermal behavior of prepared Eu-doped glasses with various molar ratios of the TeO_2_/GeO_2_ (Te0Ge to Te35Ge) was investigated in the temperature range from 100 to 800 °C (Figure 2). Additionally, the values of onset glass transition (T_g_) and crystalization in maximum (T_x_) temperatures were given in Table 1. It is seen that with the substitution of TeO_2_ with GeO_2_, the value of T_g_ temperature increased from 356 °C for Te0Ge to 420 °C for Te35Ge. This rising trend was also observed for glasses in the 65TeO_2_–20ZnO–5Na_2_CO_3_–5TiO_2_–5GeO_2_–1Tm_2_O [21] and (95 − x)TeO_2_–5WO_3_–xGeO_2_ systems [21]. The linear increase in T_g_ with changing TeO_2_/GeO_2_ molar ratio might be explained as follows: transition temperature corresponds to a change in viscosity, which is sensitive to the chemical compositions [40]. The bond strength of Ge-O (363 kJ/mol) [41] is stronger than Te-O (~250 kJ/mol) [42]. Moreover, the Ge-O bond lengths are shorter than Te-O bonds [32]. Therefore, a higher temperature is required to obtain the same viscosity with the replacement of TeO_2_ by GeO_2_, which increases T_g_.

**Figure 3:**
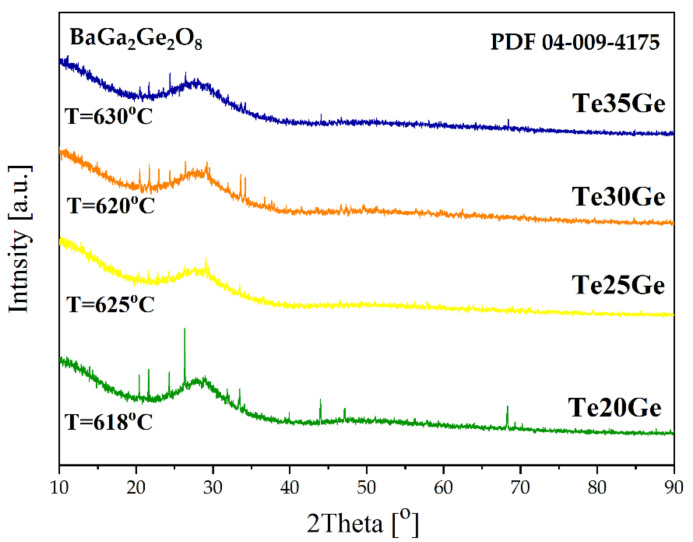
Diffraction patterns of Eu^3+^-doped Te20Ge, Te25Ge, Te30Ge, and Te35Ge glasses in maximum temperature of crystallization peaks.

It is also evident that above 15 mol% of GeO_2_, the thermal stability decreased, and in the DSC curves of the Te20Ge, Te25Ge, Te30Ge, and Te35Ge glasses, the crystallization peak was observed at 618, 625, 620, and 630 °C, respectively (Figure 2, and Table 1). To analyse the crystallization tendency of the above glasses, a heat-treatment procedure was performed on glass samples at the T_x_. The diffraction patterns of the selected heated specimens were presented in Figure 3. Besides the typical halo, several diffraction peaks were observed. Compared with the standard diffraction pattern for the crystalline phase, all diffraction lines might be assigned to the barium gallium germanium oxide BaGa_2_Ge_2_O_8_ (PDF 04-009-4175) (Figure 3). Based on the calculation of the unit cell parameters of the BaGa_2_Ge_2_O_8_ crystal structure in the Te35Ge glass, it was found that europium ions were not precipitated in the structure of barium gallium germanium oxide [32]. According to the crystallographic parameters in the reference pattern no 04-009-4175, the value of the lattice parameters lengths of the cell edges a, b, c was 9.3490 Å, 9.9030 Å, and 8.7700 Å, respectively. Calculated parameters a, b, and c of the unit cell of BaGa_2_Ge_2_O_8_ crystals in the Te35Ge sample were: a = 9.3485 Å, b = 9.9015 Å, c = 8.7500 Å.

### 3.3. Structural Studies

Several studies on the structure of tellurite, germanate, and tellurite-germanate glasses with various spectroscopy techniques have been performed, such as IR, Raman, and solid-state nuclear magnetic resonance (MAS NMR) spectroscopy [37,43,44,45,46,47,48,49,50,51,52]. The network of pure tellurite glasses can be formed by [TeO_4_] trigonal bipyramidal (btp), [TeO_3_] trigonal pyramidal (tp), and [TeO_3+1_] intermediate units. The last units are the deformed double triangular pyramid. When a cation modifier is added into the TeO_2_ glass, it breaks bridging Te–O–Te linkages, and the transformations of the [TeO_4_] → [TeO_3_] and [TeO_4_] → [TeO_3+1_] have been placed [53,54]. The structural units of the network of pure germanate glasses can be formed with tetrahedra [GeO_4_], trigonal bipyramid/square pyramid [GeO_5_], and octahedra [GeO_6_] [48].

To investigate the effect of composition variation on the structure of glass, and the local environment around Eu^3+^ ions, the MIR, and Raman spectra studies of glasses have been investigated.

#### 3.3.1. MIR Spectra

**Figure 4:**
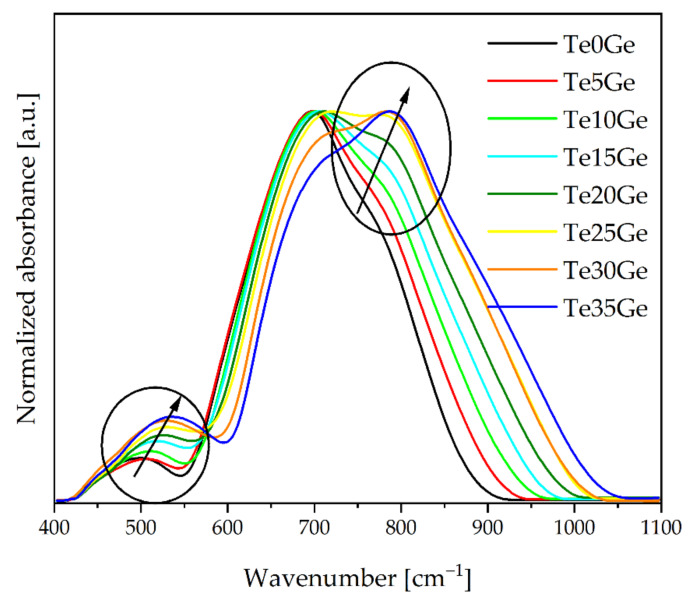
MIR spectra of Eu^3+^-doped glasses with various TeO_2_/GeO_2_ molar ratios.

MIR spectra of tellurite and tellurite-germanate glasses in the 1100–400 cm^−1^ range were shown in Figure 4. The two broad bands were observed in the range of 1100–600 cm^−1^ and 600–400 cm^−1^. As shown in Figure 4, the maxima and intensity of the bands strongly depend on the glass composition. Additionally, the position of bands in the above-mentioned ranges was shifted toward higher frequencies, and this behavior demonstrated the change in the strength of the chemical bonds due to the addition of the GeO_2_ [12]. The differences in the MIR spectra of glasses related to their composition occurred in the medium range (1100–400 cm^−1^, in elliptic selections) characteristic of stretching and deformation vibrations of different tellurites and germanates as well gallate units. The overlapping bands due to the vibrations of different bonds makes the FTIR and Raman bands broader. Additionally, the changes in the b building units’ bond angles/bond lengths due to network modifications also make the bands broader [48].

**Figure 5:**
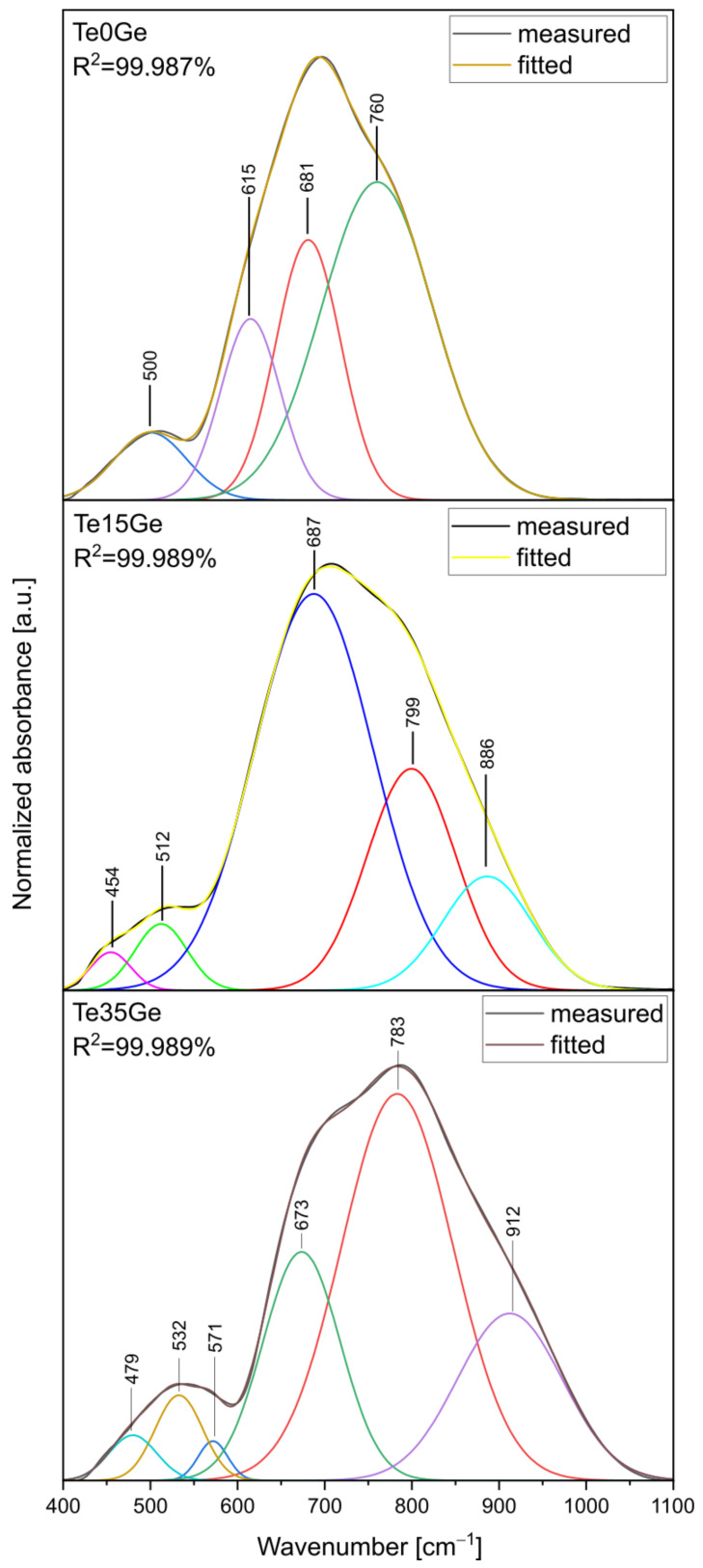
MIR spectra decomposition of the Te0Ge, Te15Ge, and Te35Ge glasses doped with Eu^3+^.

To fully comprehend the changes in the network of the glasses, the MIR spectra of the Te0Ge, Te15Ge, and Te35Ge glasses have been decomposed and shown in Figure 5. The assignment of the components bands was presented in Table 2.

The decomposed MIR spectra of the tellurite (Te0Ge) and tellurite-germanate (Te15Ge, Te35Ge) glasses showed four, five, and six bands, respectively. The bands at 500 cm^−1^, 615 cm^−1^, 681 cm^−1^, and 760 cm^−1^ were observed in the decomposed spectrum of the Te0Ge glass. There were bands at 454 cm^−1^, 512 cm^−1^, 687 cm^−1^, 799 cm^−1^, and 886 cm^−1^ in the decomposed MIR spectrum of the Te15Ge glass. The component bands at 479 cm^−1^, 532 cm^−1^, 571 cm^−1^, 673 cm^−1^, 783 cm^−1^, and 912 cm^−1^ presented in the decomposed MIR spectrum of the Te35Ge glass.

The bands at 450 cm^−1^, and 500–530 cm^−1^ were due to bending vibrations of Te–O–X, (X = Te, Ge, Ga), which was formed by corner-sharing of [TeO_4_]tbp, [TeO_3_]tp, [TeO_3+1_], [GeO_4_], [GeO_5_] [GeO_6_], and [GaO_4_], [GaO_6_] units [55]. The band at 570 cm^−1^ was attributed to the stretching vibrations of Ge(IV)–O–Ge(VI) bonds in [GeO_6_] octahedra [48]. The band at 615 cm^−1^ might be associated with the stretching vibrations of [TeO_4_]tbp units with bridging oxygen [56]. The band at 670–680 cm^−1^ was assigned to trigonally coordinated tellurium ions [TeO_3_]tp/[TeO_3+1_] [56]. The symmetrical stretching vibrations of [TeO_3_]tp/[TeO_3+1_] units with non-bridging oxygens (NBO) were detected in the range of 760–790 cm^−1^ [56]. The band at ~880–920 cm^−1^ appeared due to the asymmetrical stretching vibrations of the Ge-O-Ge connecting [GeO_4_] tetrahedra [57]. It was observed that the addition of GeO_2_ created bands overlapping with TeO_2_ and GaO_2_ bands.

It might be seen in the Te15Ge and Te35Ge samples that the addition of the GeO_2_ (up to 15 mol%) caused the appearance of additional bands at 455 cm^−1^ and 479 cm^−1^ due to bending vibrations of Te–O–Ge bonds. Moreover, IR bands at ~886 cm^−1^ and ~909 cm^−1^ were found for the Te15Ge, Te35Ge glasses. These bands could be due to the [GeO_4_] units attributable to the asymmetric stretching vibrations of Ge–O–Ge. The IR band associated with trigonally coordinated tellurium ions [TeO_3_]tp/[TeO_3+1_] (band at 670–680 cm^−1^) was found for all glasses. Still, its intensity increased as the GeO_2_ was increased to 15 mol% and decreased above 15 mol% GeO_2_. The intensity of the symmetrical stretching vibrations of [TeO_3_]tp/[TeO_3+1_] units with non-bridging oxygens (band at 760–790 cm^−1^) was changed in the case of GeO_2_ addition. For the 15 mol%, GeO_2_ showed a decrease in the intensity of this band. On the other hand, above 15 mol% GeO_2_, the band’s intensity at 799 cm^−1^ was increased. Moreover, in the decomposed MIR spectrum of the Te35Ge glass, a band appeared at ~571 cm^−1^, indicating the [GeO_6_] octahedral units existed in the glass network (Figure 5). This suggests that the glass network modification has occurred in the tellurite and germanate subnetworks due to the TeO_2_/GeO_2_ molar ratio variation. Based on presented results, the compositional evolutions of the glass network can be explained as follows: with increasing GeO_2_ content from 15 mol% up to 35 mol%, the compositional evolution was followed by a conversion of Ge(IV) to Ge(VI), and network continuity broke down with the formation of more significant numbers of NBO. Comparing our investigation results on the coordination number of germanium ions with data for germanate and TeO_2_-GeO_2_ based glasses, there was a germanate anomaly in the present glass systems. According to the adopted model of the mechanism, the germanate anomaly is the change of germanium from 4-coordinated to higher coordinated species, five-fold and six-fold Ge [58].

#### 3.3.2. Raman Spectra

**Figure 6:**
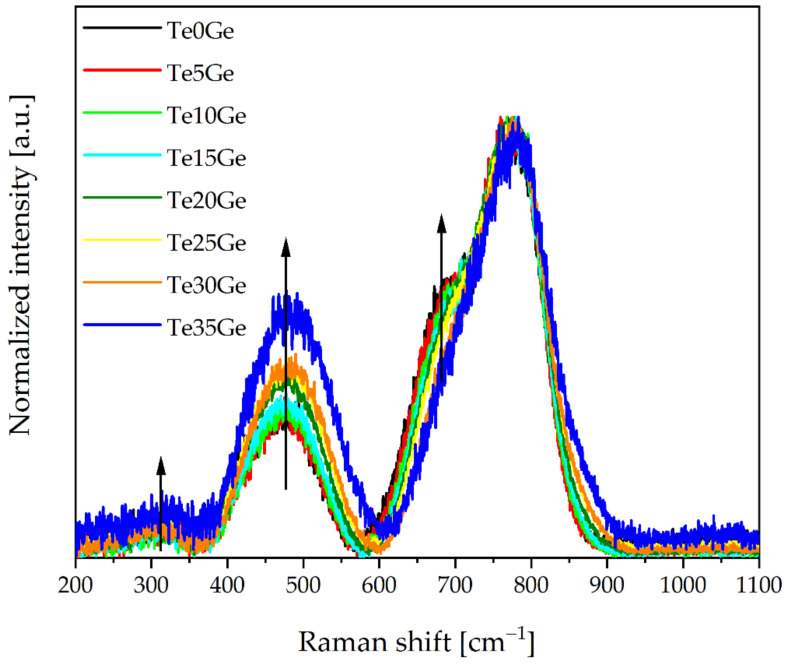
Raman spectra of Eu^3+^-doped glasses with various TeO_2_/GeO_2_ molar ratios.

The Raman spectra of Eu^3+^-doped glasses in the 1100–200 cm^−1^ range were presented in Figure 6. The obtained results are very similar to those published in [27,39]. All Raman spectra have identical features: four-banded maximums at around 320, 460, 800, and 850 cm^−1^, respectively. Differences between the intensity of the bands in Raman spectra of glasses with the increased amount of germanium dioxide seen in each region of Raman shift.

**Figure 7:**
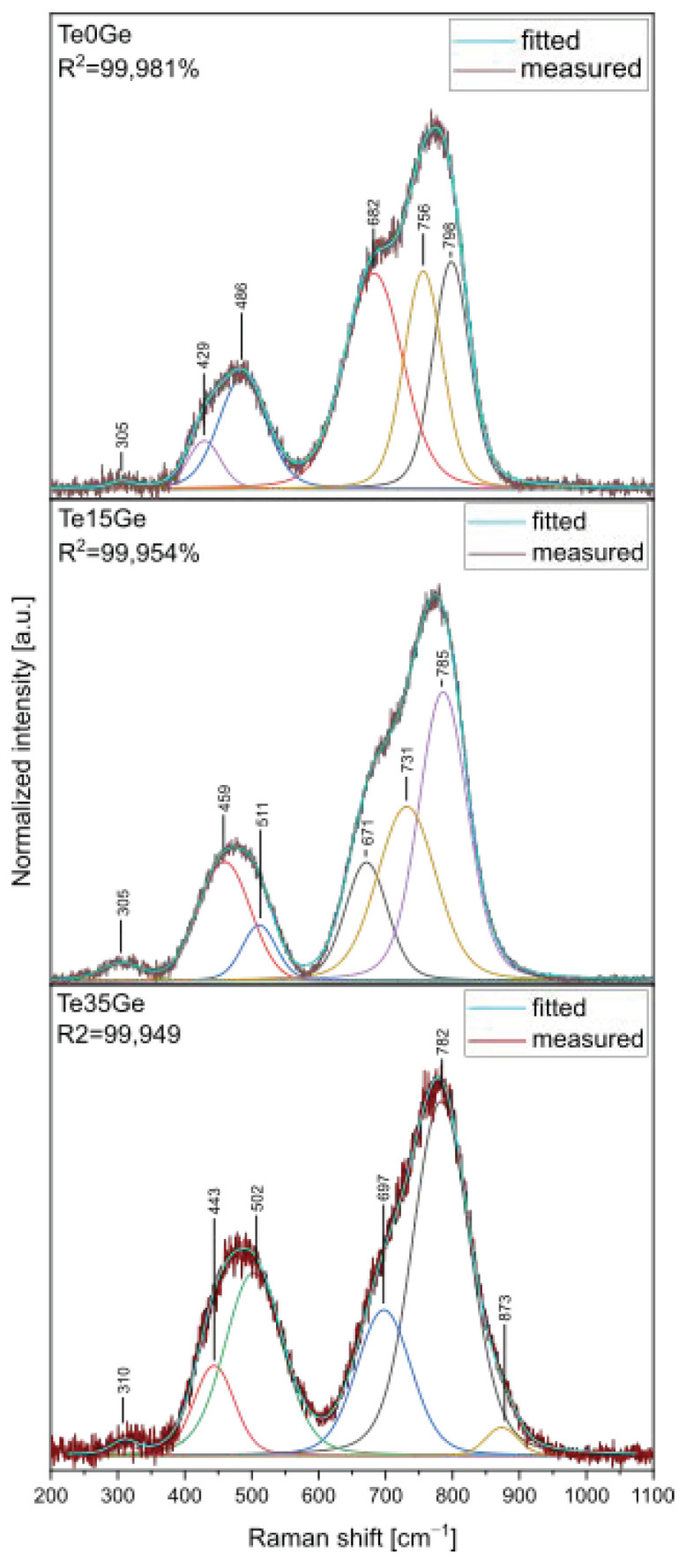
Raman spectra decomposition of the Te0Ge, Te15Ge, and Te35Ge glasses doped with Eu^3+^.

Raman spectra decompositions of Te0Ge, Te15Ge, and Te35Ge glasses doped with Eu^3+^ were carried out to evaluate the contribution of various structural units of the Raman spectra in glasses, and they were shown in Figure 7. Raman spectra were first smoothed out and then decomposed. The parameters and assignments of the bands were listed in Table 3. Obtained results showed that there were six bands in the Raman spectra of Te0Ge, Te15Ge, and Te35Ge glasses doped with Eu^3+^, at 305–310 cm^−1^, 420–460 cm^−1^, 480–510 cm^−1^, 670–700 cm^−1^, 730–750 cm^−1^, and 780–870 cm^−1^.

The band in the 305–310 cm^−1^ range can be associated with the bending vibrations of the Te–O–X bridges, where X = Te, Ga, Ge [59]. The band in the 420–460 cm^−1^ range was related to symmetric stretching vibrations of Te–O–Te bridges formed by corner-sharing of [TeO_4_]tbp, [TeO_3+1_] polyhedra, and [TeO_3_]tp units [60]. Moreover, the band in the 420–460 cm^−1^ range overlapped with symmetric stretching vibrations of Ge–O–Ge in 4-membered GeO_4_ rings [48]. The band in the 480–510 cm^−1^ range was associated with bending vibrations of the Te–O–Te bridges in [TeO_4_]tbp and [TeO_3_]tp/[TeO_3+1_] [61] units as well as with symmetric stretching vibrations of Ge–O–Ge in 3-membered GeO_4_ rings [62]. Additionally, in the 400–550 cm^−1^ range in the Raman spectra of gallate glasses, bands assigned to the bending vibration of the Ga–O–Ga bond appeared [40,60]. The band in the 670–700 cm^−1^ range came from asymmetrical stretching vibrations of Te–O–Te between [TeO_4_]tbp and [TeO_3_]tp/[TeO_3+1_]units [59]. The band in the 730–750 cm^−1^ range was related to the stretching vibrations of the [TeO_3+1_] units and TeO_3_^2–^ trigonal pyramids (tp’s) with three terminal oxygen atoms [63] or symmetrical stretching vibrations of the Ge-O^-^ of Ge^(1)^ unit [64]. The band in the 780–800 cm^−1^ range was related to the stretching vibrations of Te–O^−^ in [TeO_3_]tp and [TeO_3+1_] units and vibrations of the continuous [TeO_4_]tbp network [65]. This band could be overlapped with antisymmetric stretching vibrations of Ge-O^−^ of Ge^(2)^ units [64]. The band at around 870 cm^−1^ was an assignment to the symmetrical stretching vibrations of Ge-O^−^ in Ge^(3)^ units [48]. Moreover, the range of the 670–870 cm^−1^ in the Raman spectra might be ascribed to the stretching vibration of O–Ga–O [40,66]. What’s more, the band in the 620–650 cm^−1^ range characteristic for symmetric stretching vibration of Ge–O–Ge bonds in [GeO_6_] octahedral units was not detected in the decomposed Raman spectra (band at 620–650 cm^−1^) [61,66]. Due to the overlapping of different component bands in the decomposed Raman spectra in the analyzed multicomponent glass system, the correct interpretation of the changes in the integral intensities is impossible. Considering the above, the authors limited the interpretation of decomposed Raman spectra to qualitative analysis. The network of glasses with GeO_2_ content consists of [TeO_4_]tbp, [TeO_3_]tp/[TeO_3+1_] units. However, Ge^(3)^ units appeared in the Te35Ge glass, not found in the Te15Ge glass.

### 3.4. Optical Studies

#### 3.4.1. Refractive Index

**Figure 8:**
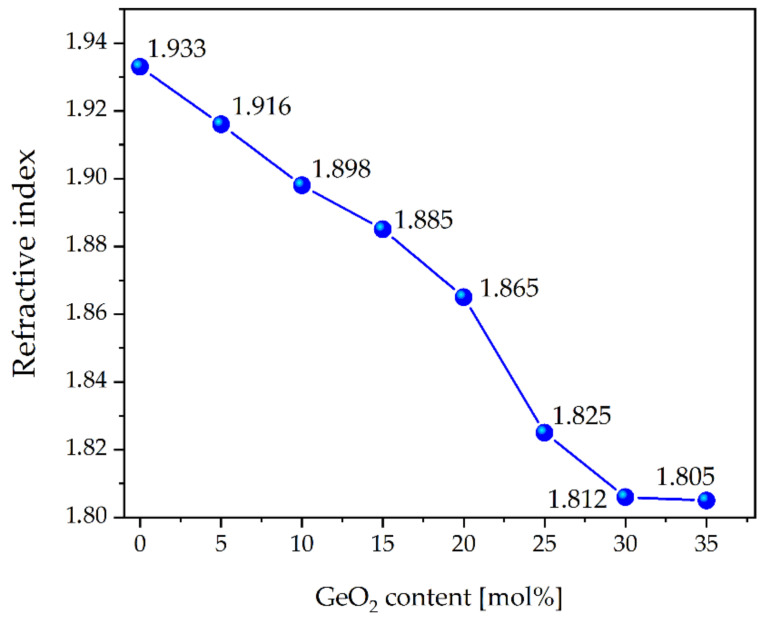
Refractive index of Eu^3+^-doped tellurite glasses.

The value of the refractive index, measured for 632.8 nm wavelength (Figure 8), decreases with increasing GeO_2_ content from 1.933 for Te0Ge glass to 1.805 nm for Te35Ge glass. This trend observed with an increase in GeO_2_ content might be consistent with electronic polarizability for germanium, and tellurium ions [21]. As a result, the polarizability (α) of tellurium ion is larger than the other cations (α_Te_ = 1.595 Å for Te^4+^, α_Ge_ = 0.137 Å for Ge^4+^, α_Ga_ = 0.195 Å for Ga^3+^, (α_Zn_ = 0.283 Å for Zn^2+^, α_Ba_ = 1.595 Å for Ba^2+^, α_Na_ = 0.181 Å for Na^+^, α_Eu_ = 1.12 Å for Eu^3+^) [67]. Obtained data are compatible with those reported in the literature [32,68].

#### 3.4.2. Excitation Spectra and Phonon Sideband Analysis

**Figure 9:**
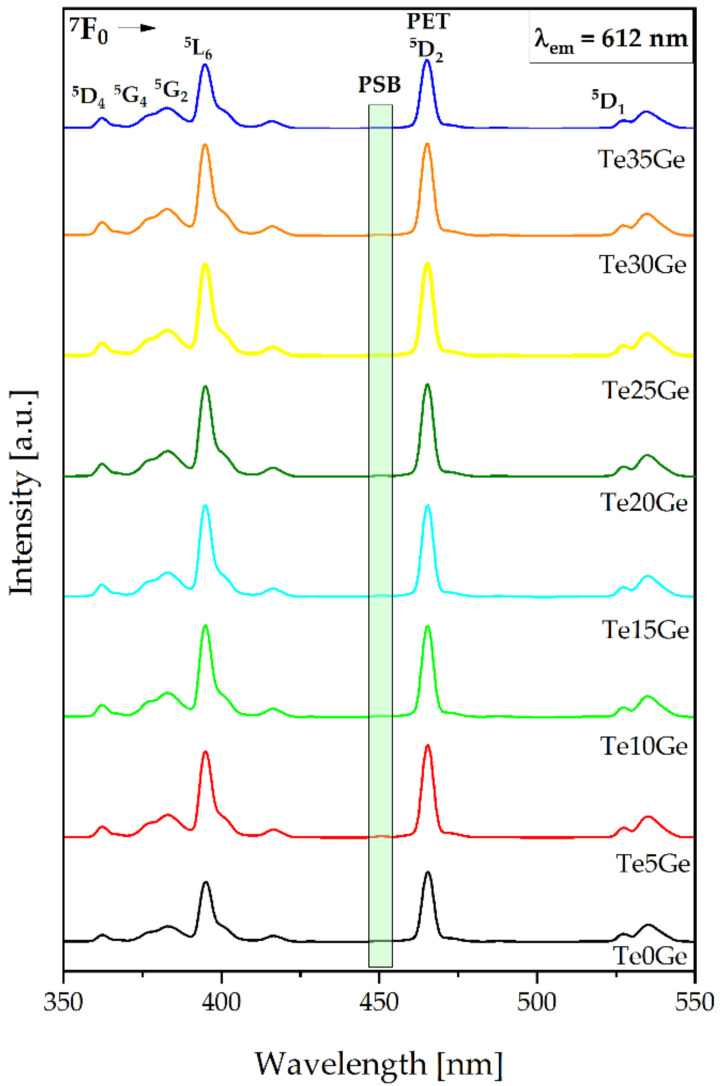
PLE spectra of Eu^3+^-doped glasses with various TeO_2_/GeO_2_ molar ratios.

**Figure 10:**
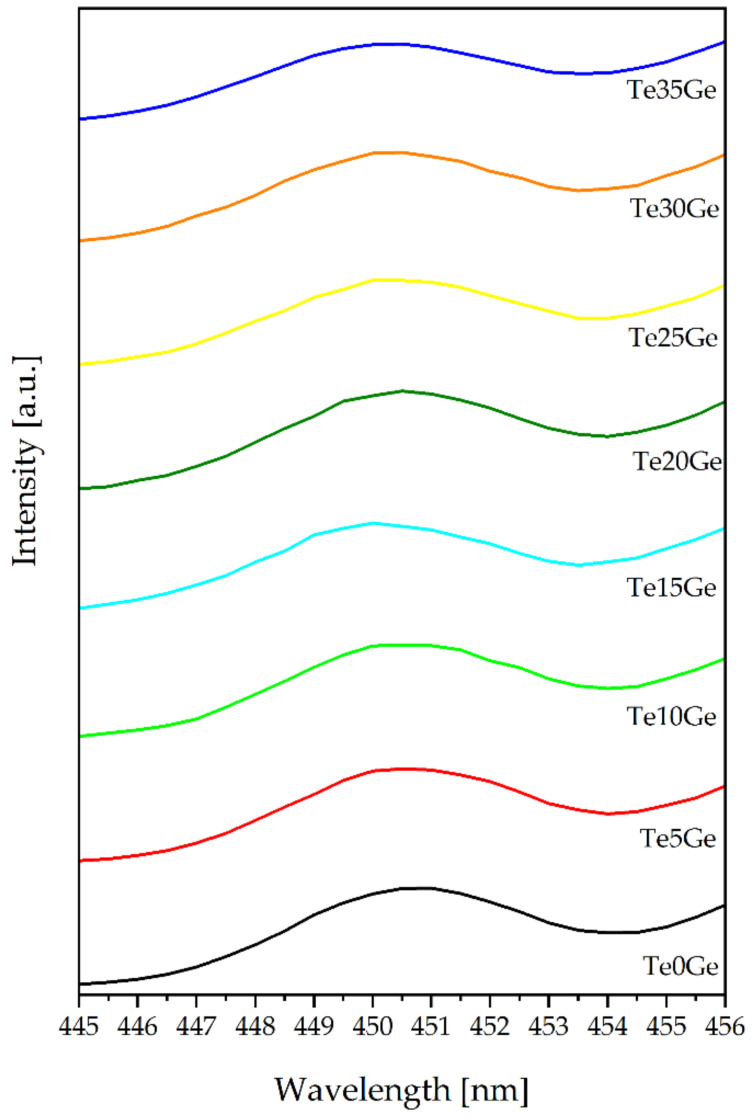
Phonon sidebands (PSB) of Eu^3+^ ions in glasses with various TeO_2_/GeO_2_ molar ratios.

The excitation spectra of the Eu^3+^-doped glasses with various TeO_2_/GeO_2_ molar ratios were recorded in the 350–550 nm by monitoring the emission at 611 nm corresponding to the ^5^D_0_ → ^7^F_2_ transition. The results were presented in Figure 9. Several bands are assigned to the following transitions: ^7^F_0_ → ^5^D_4_ (362 nm), ^7^F_0_ → ^5^G_4_ (376 nm), ^7^F_0_ →^5^G_2_ (383 nm), ^7^F_0_ → ^5^L_6_ (395 nm), ^7^F_0_ → ^5^D_2_ (465 nm), and ^7^F_0_ → ^5^D_1_ (527) [69]. Among various transitions, at 465 nm was prominent. In Figure 9, the phonon sideband (PSB) associated with the transition of ^7^F_0_ →^5^D_2_ might be observed. Additionally, the excitation spectra from 445 to 456 cm^−1^ range were shown in Figure 10. PSB analysis is a useful tool to determine the phonon energy of the glass host and the local environment around the Eu^3+^ ions in the glass network. Based on the difference between the positions of the PSB and PET bands, it might be possible to calculate the phonon energy associated with the maximum energy of the vibrational mode around the europium ions [70]. The evaluated phonon energies for the presented glasses increased with the addition of the GeO_2_ content and were as follows: 699 cm^−1^ (Te0Ge) glass, 714 cm^−1^ (Te5Ge) glass, 702 cm^−1^ (Te10Ge) glass, 716 cm^−1^ (Te15Ge) glass, 705 cm^−1^ (Te20Ge) glass, 726 cm^−1^ (Te25Ge) glass, 746 cm^−1^ (Te30Ge) glass, and 749 cm^−1^ (Te35Ge) glass (Table 4). The phonon energies values were smaller than heavy metal oxide glasses published in the literature [71,72]. The phonon energies for glasses from the excitation spectrum PSB-PET agreed with the Raman spectra of glasses (Figure 7 and Table 3).

#### 3.4.3. Emission Spectra Analysis

**Figure 11:**
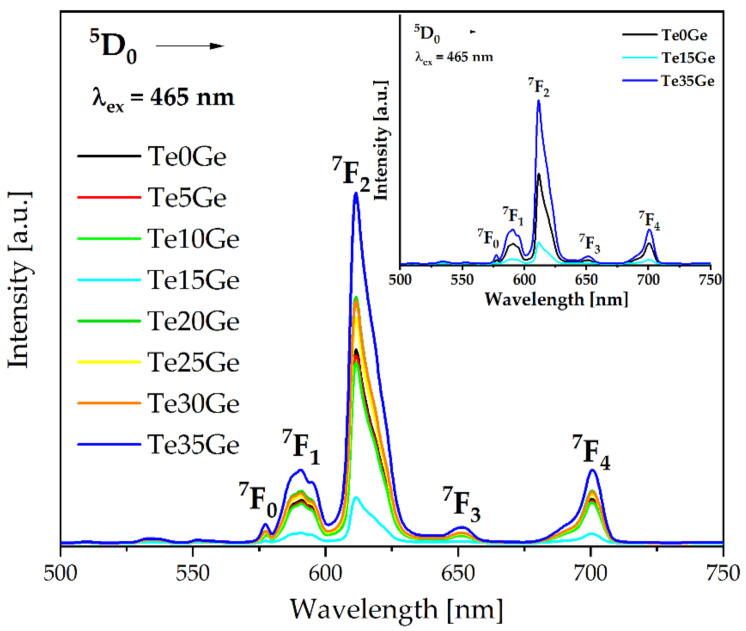
PL spectra of Eu^3+^ ions in glasses under 465 nm excitation. The PL spectra selected glasses (inset).

The emission spectra (PL) of all the Eu^3+^-doped glasses were measured under 465 nm excitation. Due to the relaxation from the ^5^D_0_ state, the intense emission bands were obtained in the 550–750 nm range (Figure 11). The emission bands at 577 nm, 590 nm (orange), 611 nm (red), 650 nm, and 700 nm correspond to the transitions ^5^D_0_ → ^7^F_0_, ^5^D_0_ → ^7^F_1_, ^5^D_0_ → ^7^F_2_, ^5^D_0_ → ^7^F_3_, and ^5^D_0_ → ^7^F_4,_ respectively. The obtained emission spectra are in accordance with glasses in the (60 − x)TeO_2_–10K_2_O–10P_2_O_5_–10B_2_O_3_–10ZnF_2_–xEu_2_O_3_ (x = 0.05, 0.1, 0.2, 0.4, 0.8 and 1.6 mol%) [71], (35 − x)SiO_2_–25B_2_O–10Na_2_O–15NaF–15CaF_2_–xEu_2_O_3_ (x = 0.1, 0.5, 1.0, 1.5 and 2.0 mol%) [73], and (30 − x)B_2_O_3_–30TeO_2_–16ZnO–10ZnF_2_–7CaF_2_–7BaF_2_–xEu_2_O_3_ (where x = 0.1, 0.25, 0.5, 1.0, 2.0, and 3.0 in wt%) [74] systems. As might be seen in Figure 11, the intensity of the bands in the emission spectra (PL) of glasses depends on the GeO_2_ content. For the glasses with up to 15 mol% of GeO_2_, an increase in the intensity of the bands was observed. However, the further increase in the GeO_2_ content caused a decrease in the intensity of the bands of the PL spectra.

**Figure 12:**
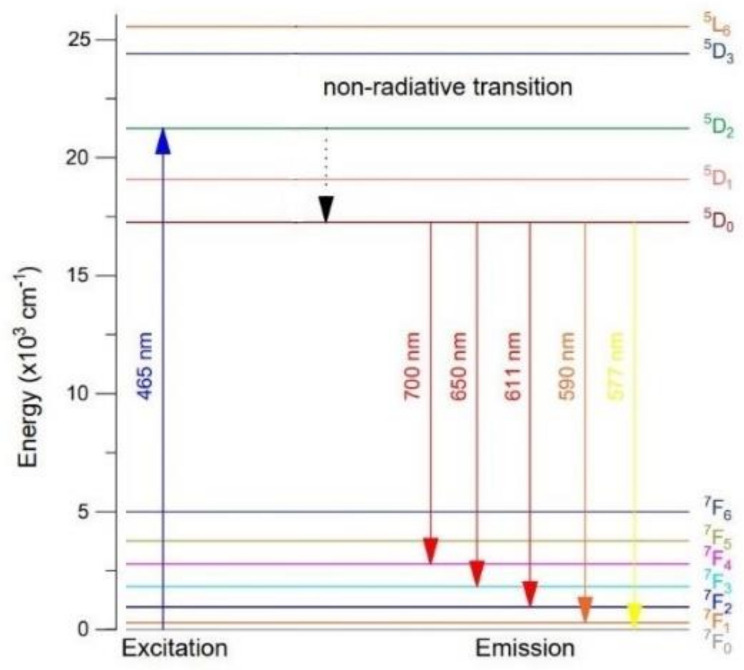
Energy level diagram of Eu^3+^ ions in the investigated glasses.

The possible energy level diagram of the europium ions in the tellurite and tellurite-germanate glasses with various TeO_2_/GeO_2_ molar ratios was presented in Figure 12. The europium ions were excited at 465 nm wavelengths to the upper levels of ^5^D_2_, respectively. The pumped electrons relaxed to the lowest excited level ^5^D_0_ by non-radiative transition and radiatively relaxed to the ground state ^7^F_J_ (J = 0, 1, 2, 3, and 4). The non-radiative multiphonon relaxation to the ^5^D_0_ level occurred when the europium ions were excited to levels above this level (^5^D_0_). Afterwards, intense emission bands appeared due to ^5^D_0_ → 7F_0_, 7F_1_, 7F_2_, 7F_3_, 7F_4_ transitions.

It is well-known that ^5^D_0_ → ^7^F_1_ transition in the orange region with ΔJ = 1 has been identified as magnetic-dipole (MD) transition. The following red emission at 611 nm (^5^D_0_ → ^7^F_2_) is considered as a hypersensitive transition owing to the selection rule ΔJ = 2 and has been classified as the electric-dipole (ED) transition. The changes influence the intensity of this transition in the environment surrounding europium ions. The domination of the ED transition in emission spectra is direct proof that europium ions are situated in non-centrosymmetric sites. If Eu^3+^ ions occupy centrosymmetric sites, the probability of the MD transition occurrence is higher than the ED one. The proximity of the europium environment to the centrosymmetry might be characterized by the fluorescence intensity ratio (R) of the electric (^5^D_0_ → ^7^F_2_) to magnetic (^5^D_0_ → ^7^F_1_) dipole transition intensity. Moreover, the R ratio indicates the strength of covalent/ionic bonding between the europium ions and the surrounding ligands [74].

**Figure 13:**
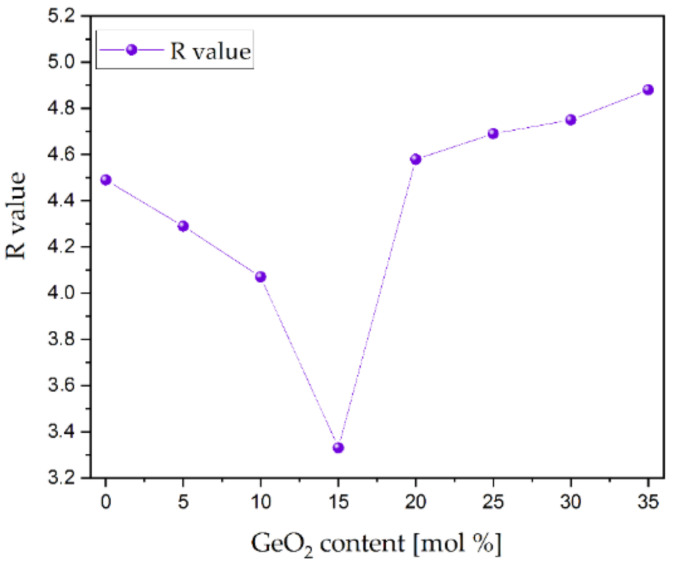
R values vs. GeO_2_ content.

The fluorescence intensity ratio (R) for the Eu^3+^-doped glasses with various TeO_2_/GeO_2_ molar ratios are shown in Figure 13. The high fluorescence intensity ratio (R) values indicated that europium ions occupy the low symmetry sites [63,75,76]. Hence, the high R values indicated the covalent nature of the Eu-O bond [77,78]. It was also observed that the asymmetry was decreased with an increase in GeO_2_ content up to 15 mol%. However, the addition of germanium dioxide above 15 mol% increased the asymmetry of Eu^3+^ ions.

The obtained values of fluorescence intensity ratio for the Eu^3+^ ions in the tellurite and the tellurite-germanate glasses supported the hypothesis of Wang et al. [79] that the strongly depolymerized network in oxide glasses enhances the regularity of the sites occupied by the lanthanide ions. According to the crystal-chemistry model, the local environments of the rare earth cations are mainly influenced by three factors: (a) the number of coordinating anions around the rare-earth cation; (b) the size of the rare-earth cation, and (c) the degree of the depolymerization of the network. The regularity of the site occupied by the rare-earth cation increases as polymerization decreases. In the Te0Ge and Te15Ge glasses, the Eu^3+^ ions can coordinate [TeO_3_]^2−^ anions (MIR results). For Te35Ge glass, the presence of TeO_3_^2−^ [GeO_6_]^2−^, and Ge-O^−^ of the Ge^(3)^ unit is responsible for disturbing the symmetry in the local framework of Eu^3+^, and thereby the probability of the ^5^D_0_ → ^7^F_2_ transition can be enhanced in glasses up to 15 mol%GeO_2_, and a decrease in R/O ratio values above 15 mol% of GeO_2_ was observed.

#### 3.4.4. Luminescence Decay Analysis

**Figure 14:**
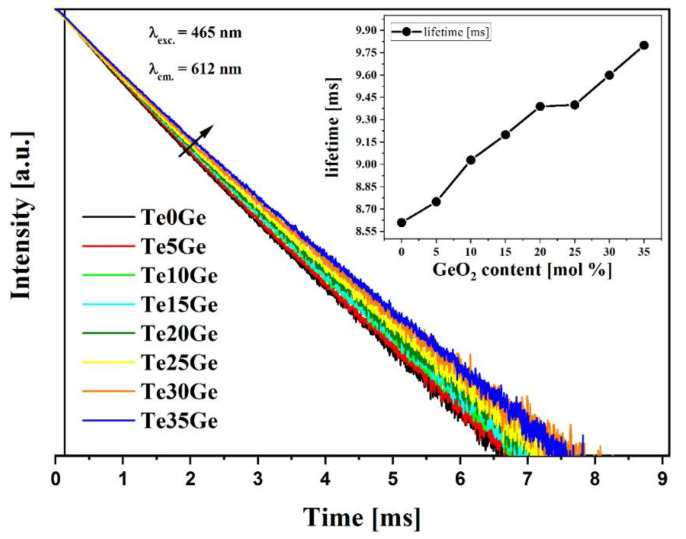
Luminescence decay curves of the ^5^D_0_ excited state of Eu^3+^ ions in studied glasses.

The luminescence decays from the ^5^D_0_ state were recorded for λ_em_ = 612 nm (^7^F_0_ →^5^D_2_) using λ_exc_ = 465 nm (^7^F_0_ → ^5^D_2_). The decay curves registered for glasses were well-fitted by the single-exponential I(t)/I_0_ = Aexp(-t/τ_m_) function, where τ_m_ corresponds to luminescence decay time [80]. Obtained results were presented in Figure 14. The calculated luminescence lifetimes in glasses were equal to 8.61 ms (Te0Ge), 8.75 ms (Te5Ge), 9.03 ms (Te10Ge), 9.20 ms (Te15Ge), 9.39 ms (Te20Ge), 9.40 ms (Te25Ge), 9.60 ms (Te30Ge), and 9.80 ms (Te35Ge) what might be seen in Figure 14. Values of the luminescence lifetimes for the ^5^D_0_ state of europium ions increased when GeO_2_ in the glass composition replaced TeO_2_. It was confirmed that the lifetime for the ^5^D_0_ state of europium increases with increasing phonon energy in heavy metal oxide glasses [66]. A similar situation was observed for Eu^3+^ ions in our studies. [81]. The measured lifetime for the ^5^D_0_ state of Eu^3+^ enlarged with the increasing phonon energy of studied glasses due to GeO_2_ addition. The increase in a lifetime ^5^D_0_ state of Eu^3+^ with increasing phonon energy can be explained with regard to the energy gap law of europium ions. The energy gap between excited level ^5^D_0_ and lower level ^7^F_6_ is large and has ∆E = 12,500 cm^−1^. Due to non-radiative relaxation, rates for rare-earth are negligibly low. The value of the order of multiphonon relaxation for the ^5^D_0_ state of Eu^3+^ amount 16 in our glasses. The rates of multiphonon relaxation processes at ten rows are negligible [81].

### 3.5. Optical Fiber

In recent years, significant progress has been made in expanding the spectral range of tellurite-based fiber lasers generating at wavelengths above 2 µm. A detailed review on the state of the art of Nd^3+^-, Er^3+^-, Tm^3+^-, Ho^3+^-doped tellurite glass fiber was presented by E. A. Anashkina [82]. In this section of the manuscript, the results of the glass fiber draw were demonstrated. The glass preform was made from the Te35Ge glass composition.

**Figure 15:**
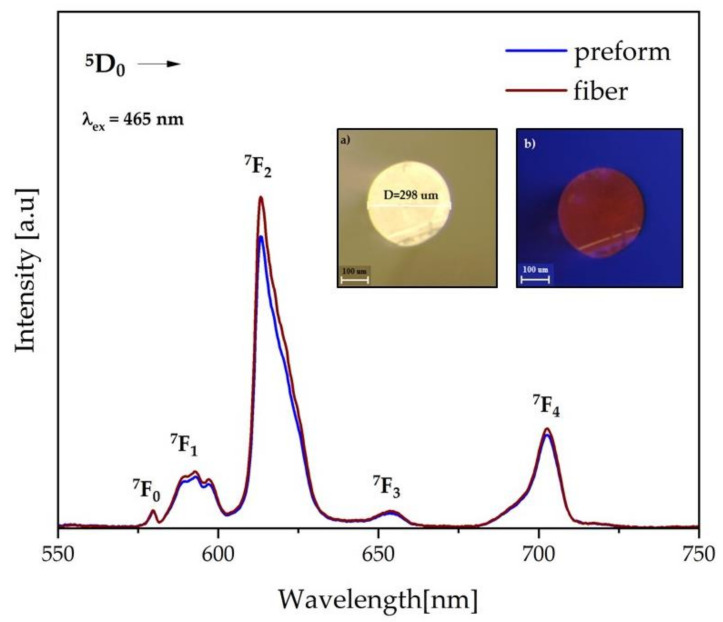
PL spectra of the optical fiber recorded under 465 nm excitation. Inset: microphotographs of the glass fiber in (**a**) white light (with fiber diameter), (**b**) deep-blue light (with highlighted orange-reddish emission of the fiber).

Figure 15 presents the result of the PL spectra of the Eu^3+-^doped glass fiber under the 465 nm excitation. The bands located on the spectra were adequate to the ones given in Figure 11, and they indicate the same transitions: ^5^D_0_ → ^7^F_0_ (577 nm), ^5^D_0_ → ^7^F_1_ (590 nm), ^5^D_0_ → ^7^F_2_ (611 nm), ^5^D_0_ → ^7^F_3_ (650 nm), and ^5^D_0_ → ^7^F_4_ (702 nm). In the inset of Figure 15, the microphotographs of the fiber surface in the white light (a) and the deep-blue light (b) are given. As a result of all transitions above, the fiber displays a rich orange-reddish emission (Figure 15b.)

## 4. Discussion

Novel tellurite and tellurite-germanate glasses with a GeO_2_ content of up to 35 mol% were obtained by the melt quenching method.

Their thermal stability was analyzed to fulfill optical fiber drawing requirements according to the glass compositions. The DSC curves showed a rise in transition temperature with an increase in GeO_2_ content in the glass composition from 356 to 420 °C. Moreover, the addition of germanium oxide above 15 mol% content caused a decrease in the thermal stability of the glasses. A crystallization peak was detected in the DSC curves of the glasses with 20, 25, 30, and 35 mol% of GeO_2_. After heat treatment at the maximum temperature, the exothermic peak, BaGa_2_Ge_2_O_8_ crystal phase, appeared (Figure 3). The increased tendency towards crystallization agrees with structural studies (MIR, Raman spectroscopy).

The change in the network of glasses with various TeO_2_/GeO_2_ molar ratios had an important impact on the local environment of Eu^3+^ ions. This might be explained considering the field strengths and electronegativity of the network-modifying ions. According to Rada et al. [83], the presence of multiple oxides in the glass increased the tendency of the network formers to attract the structural units for compensation of the charge yield. In Figure 5, the component band assigned to the symmetrical stretching vibrations of [TeO_3_]tp units with non-bridging oxygens (NBO) was found in decomposed MIR spectrum of the Te0Ge glass. With the increase of the GeO_2_ up to 15 mol%, the number of symmetrical stretching vibrations of [TeO_3_]tp/[TeO_3+1_] units with non-bridging oxygens decreased. This suggests that modifier ions strongly affinity towards these groups containing NBO. The higher field strength of europium ions than Zn^2+^, Ba^2+^, and Na^+^ ions [84] was reasonable to increase the crosslinking of the Te15Ge glass network compared to the Te0Ge glass. Based on the decomposed MIR and Raman spectra, the presence of [TeO_4_]tbp, [TeO_3_]tp, and [TeO_3+1_], [GeO_4_], Ge^(1)^, Ge^(2)^, and [GaO_4_] was found in the glass network of the Te15Ge glass.

On the other hand, in the network of the Te35Ge glass, there appeared to be negatively charged [GeO_6_]^2−^ and Ge-O^−^ of the Ge^(3)^ units [85]. In glasses with GeO_2_ content above 15 mol%, the europium ions were surrounded by a concentration of negative charge from NBO atoms in tellurite and germanium units to the neutrality of the structure. However, PSB associated with the ^7^F_0_→^5^D_2_ PEB indicated the coupling of lower vibrational modes of [TeO_4_]tbp, [TeO_3_]tp, and [TeO_3+1_] units to the Eu^3+^ ions in the Te0Ge, Te5Ge, Te10Ge, and Te15Ge glasses. In contrast, europium ions in the Te30Ge and Te35Ge glasses were also coupled with higher modes of germanate units (Table 4). The change in the structure and the local environment of Eu^3+^ ions resulted in a change in the luminescence properties of studied glasses.

Moreover, the measured lifetime for the ^5^D_0_ state of Eu^3+^ increased with the phonon energy of studied glasses. The glass composition characterized by the most extended lifetime of europium ions was used as a core material to draw optical fiber. It is also worth pointing out that the value of the refractive index of glasses varies from 1.933 to 1.805 with increasing GeO_2_, which is helpful to adjust glass composition for fiber cladding in the future. The broad refractive index change was consistent with the larger polarizability of the tellurium ion than the other cations [67].

## 5. Conclusions

The Eu^3+^-doped glasses in the TeO_2_-xGeO_2_-Ga_2_O_3_-ZnO-BaO-Na_2_O system (x = 0–35 mol%) were investigated to explore their potential application as a host material for RE doping and optical fiber drawing. The thermal, structural, and optical properties have been considered as the main factors. The thermal evaluation showed high glass stability, although a crystallization peak appeared above 15 mol% of GeO_2_. This was correlated with the MIR and Raman results, which indicated that the conversation of the germanium ions from tetrahedral to octahedral coordination is promoted. It is concluded that glasses with 20–35 mol% germanium dioxide content possess a weaker network than more polymerized glasses with 5–15 mol% of GeO_2_. The above structural changes are also seen when analyzing fluorescence intensity ratio (R) for the Eu^3+^ ion, and its asymmetry decreased with the GeO_2_ content up to 15 mol% and returned to 20 mol%. Despite the above changes, the luminescence behavior of Eu^3+^-doped tellurite and tellurite-germanate glasses showed the differences in emission intensity and the continuous growth of the lifetime, which can be explained by the large energy gap of Eu^3+^ ions in the glass matrix, having in mind the increasing phonon energy of studied glasses.

In that aspect, the correlation between the phonon energy of glasses and GeO_2_ content was determined based on PSB analysis. The characteristic hypersensitive emission bands corresponding to ^5^D_0_ → ^7^F_J_ (J = 1, 2) transitions in the europium ions showed the R-value increase for the glasses with GeO_2_ content up to 15 mol%. In contrast, the R factor was reduced for the glasses with a higher concentration of GeO_2_.

Glass with 35 mol% of GeO_2_ was used for optical preform and fiber fabrication. Both are achieved by intensive luminescence originating from Eu^3+^ ions, which conformed to the readiness of this glass system for active optical fiber development.

## Figures and Tables

**Table 1:** The studied samples’ glass transition (T_g_) and crystalization (T_x_) temperatures.

Glass	T_g_ (±1 °C)	T_x_ (±1 °C)
Te0Ge	356	-
Te5Ge	361	-
Te10Ge	368	-
Te15Ge	375	-
Te20Ge	380	618
Te25Ge	395	625
Te30Ge	410	620
Te35Ge	420	630

**Table 2:** Assignment of MIR bands of the Te0Ge, Te15Ge, and Te35Ge glasses doped with Eu^3+^ [48,55,56,57,58].

Positionof the Component Bands [cm^−1^]			Assignment		
Glass					
Te0Ge	Te15Ge	Te35Ge	Te0Ge	Te15Ge	Te35Ge
-	454	479	bending vibrations of Te–O–X, (X = Te, Ge, Ga)		
500	512	532			
-	-	571	Stretching vibration of the Ge(IV)-O-Ge(IV)		
615	-	-	stretching vibrations of [TeO_4_]tbp units with bridging oxygen		
681	687	673	vibrations of trigonally coordinated tellurium ions [TeO_3_]tp/[TeO_3+1_]		
760	799	783	symmetrical stretching vibrations of [TeO_3_]tp/[TeO_3+1_] units with NBO		
	886	912	asymmetrical stretching vibrations of the Ge-O-Ge connecting [GeO_4_] tetrahedra		

**Table 3:** Assignment of component bands in Raman spectra of the selected glasses doped with Eu^3+^ [59,60,61,62,63,64,65,66].

Position of Component Band [cm^−1^]			Assignment		
Glass					
Te0Ge	Te15Ge	Te35Ge	Te0Ge	Te15Ge	Te35Ge
305	305	310	bending vibrations of the Te-O-X bridges, where X = Te, Ga, Ge		
429	459	443	symmetric stretching vibrations of Te-O-Te bridges formed by corner-sharing of [TeO_4_]tbp, [TeO_3+1_]tp polyhedra, and [TeO_3_] units; symmetric stretching vibrations of Ge-O-Ge in 4-membered GeO_4_ rings; bending vibration of the Ga-O-Ga bond		
486	511	502	bending vibrations of the Te-O-Te bridges in [TeO_4_]tbp and [TeO_3_]tp units; symmetric stretching vibrations of Ge-O-Ge in 3-membered GeO_4_ rings; bending vibration of the Ga-O-Ga bond		
682	671	697	asymmetrical stretching vibrations of Te–O–Te between [TeO_4_]tbp units, and [TeO_3+1_] units; stretching vibration of O–Ga–O		
756	731	-	stretching vibrations of the [TeO_3+1_] units and TeO_3_^2−^ trigonal pyramids (tp’s) with three terminal oxygen atoms; symmetrical stretching vibrations of the Ge-O^−^ of Ge^(1)^ unit; stretching vibration of O–Ga–O		
798	785	782	stretching vibrations of Te-O^−^ in [TeO_3_]tp and [TeO_3+1_] units; vibrations of the continuous [TeO_4_]tbp network;antisymmetric stretching vibrations of Ge-O^−^ in Ge^(2)^ units; stretching vibration of O–Ga–O		
-	-	873	symmetrical stretching vibrations of Ge-O^−^ in Ge^(3)^ units; stretching vibration of O–Ga–O		

**Table 4:** Phonon energies determined from the excitation spectra of the studied glasses.

Glass	PSB	PET	PSB-PET [cm^−1^]
Te0Ge	450.77 nm (22.184 cm^−1^)	465.44 nm (21.485 cm^−1^)	699
Te5Ge	450.53 nm (22.196 cm^−1^)	465.50 nm (21.482 cm^−1^)	714
Te10Ge	450.51 nm (22.197 cm^−1^)	465.22 nm (21.495 cm^−1^)	702
Te15Ge	449.95 nm (22.224 cm^−1^)	464.93 nm (21.508 cm^−1^)	716
Te20Ge	450.45 nm (22.200 cm^−1^)	465.22 nm (21.495 cm^−1^)	705
Te25Ge	450.02 nm (22.221 cm^−1^)	465.22 nm (21.495 cm^−1^)	726
Te30Ge	450.39 nm (22.202 cm^−1^)	465.93 nm (21.462 cm^−1^)	746
Te35Ge	450.26 nm (22.209 cm^−1^)	465.98 nm (21.460 cm^−1^)	749

## Data Availability

Not applicable.
